# Integrated application of bacterial carbonate precipitation and silicon nanoparticles enhances productivity, physiological attributes, and antioxidant defenses of wheat (*Triticum aestivum* L.) under semi-arid conditions

**DOI:** 10.3389/fpls.2022.947949

**Published:** 2022-10-25

**Authors:** El-Sayed M. Desoky, Mostafa M. Rady, Maha M. Nader, Nadeen G. Mostafa, Ahmed S. Elrys, Archana Mathai, Synan F. AbuQamar, Khaled A. El-Tarabily, Mohamed T. El-Saadony

**Affiliations:** ^1^Department of Botany, Faculty of Agriculture, Zagazig University, Zagazig, Egypt; ^2^Department of Botany, Faculty of Agriculture, Fayoum University, Fayoum, Egypt; ^3^Department of Agricultural Microbiology, Faculty of Agriculture, Zagazig University, Zagazig, Egypt; ^4^Department of Soil Science, Faculty of Agriculture, Zagazig University, Zagazig, Egypt; ^5^Department of Biology, College of Science, United Arab Emirates University, Al Ain, United Arab Emirates; ^6^Khalifa Center for Genetic Engineering and Biotechnology, United Arab Emirates University, Al Ain, United Arab Emirates; ^7^Harry Butler Institute, Murdoch University, Murdoch, WA, Australia

**Keywords:** antioxidant system, *Bacillus*, reactive oxygen species, sandy soil, silicon, wheat production

## Abstract

The use of calcium carbonate-precipitating bacteria (CCPB) has become a well-established ground-improvement technique. However, the effect of the interaction of CCPB with nanoparticles (NPs) on plant performance is still meager. In this study, we aimed at evaluating the role of CCPB and/or silicon NPs (Si-NPs) on the growth, physio-biochemical traits, and antioxidative defense of wheat (*Triticum aestivum* L.) under semi-arid environmental conditions. A 2-year pot experiment was carried out to determine the improvement of the sandy soil inoculated with CCPB and the foliar application of Si-NPs on wheat plants. We tested the following treatments: spraying plants with 1.0 or 1.5 mM Si-NPs (control = 0 mM Si-NPs), soil inoculated with *Bacillus lichenforms* (MA16), *Bacillus megaterium* (MA27), or *Bacillus subtilis* (MA34), and the interaction of individual *Bacillus* species with Si-NPs. Our results showed that soil inoculation with any of the three isolated CCPB and/or foliar application of Si-NPs at the rates of 1.0 or 1.5 mM significantly improved (*p* ≤ 0.05) the physiological and biochemical attributes as well as the enzymatic antioxidant activities of wheat plants. Therefore, the combined treatments of CCPB + Si-NPs were more effective in enhancing physio-biochemical characteristics and enzymatic antioxidant activities than the individual treatments of CCPB or Si-NPs, thus achieving the best performance in the treatment of MA34 + 1.5 mM Si-NPs. Our results demonstrated that the co-application of CCPB and Si-NPs, particularly MA34 + 1.5 mM Si-NPs, considerably activated the antioxidant defense system to mitigate the adverse effects of oxidative stress, thus increasing tolerance and enhancing the production of wheat plants in sandy soils under semi-arid environmental conditions.

## Introduction

Wheat (*Triticum aestivum* L.), belonging to the Poaceae family, is the most important cereal crop globally due to its higher content of protein, carbohydrates, vitamins, and calories than other cereal crops (Elrys et al., 2020). It is cultivated in rain-fed and irrigated areas in tropical and subtropical regions. Additionally, it is grown on approximately 200 million hectares globally, yielding about 700 Tg (10^12^ g) year^–1^ (FAOSTAT, 2020). Therefore, the global need for wheat is increasing, predominantly in developing nations with limited croplands and resources, including Egypt, which poses challenges in producing the quantities of wheat required to meet this growing demand. For instance, wheat covers around 1.40 million hectares in Egypt, producing 9.0 Tg of grain in 2019, approximately 34.5% of annual consumption (FAOSTAT, 2020).

Consequently, it is imperative to maximize wheat production, especially in soils with poor physicochemical and biological properties, such as sandy soils, which cover about 90% of the Egyptian soils (Merwad and Abdel-Fattah, 2015). Furthermore, under Egypt’s semi-arid climatic conditions, these soils provide significant prospects for agricultural expansion. Promising techniques for increasing productivity in such soils include effective agricultural bio-systems that consider the biochemical diversity of agricultural systems, their ability to reduce the negative influences of low soil fertility, and water-retaining capacity in sandy soils. However, the use of calcium carbonate (CaCO_3_) precipitating bacteria (CCPB) and silicon (Si) nanoparticles (Si-NPs) are innovative and effective technologies that improve the productivity of crops under semi-arid environmental conditions (Chaparro-Acuña et al., 2018; Desoky et al., 2021).

The precipitation of CaCO_3_ is a process in which microorganisms, mainly bacteria, provide adequate substrates, creating CaCO_3_ crystals (Chaparro-Acuña et al., 2018). The *Bacillus* group is non-pathogenic and tolerant of extreme conditions, with high concentrations of urease enzyme (Achal et al., 2015); therefore, it can potentially be used as CCPB. Urease hydrolyzes urea in soils to carbonate (CO_3_^2–^) and ammonium (NH_4_^+^). The produced NH_4_^+^ raises the pH of the solution, causing the reaction to form CaCO_3_ on the bacterial cell surface whenever there is sufficient calcium (Ca^2+^) and CO_3_^2–^ ion content in the solution (Chaparro-Acuña et al., 2018). The resulting CaCO_3_ can coat surfaces and bind various particles together (Seifan et al., 2020). CCPB is a practical approach to enhance soil quality (Chaparro-Acuña et al., 2018) and increase sand stabilization and soil hardness while decreasing soil porosity in sandy soils (Whiffin et al., 2007). Similarly, the induction of CCPB binds sand grains and enhances soil stiffness and strength (DeJong et al., 2010; Mortensen et al., 2011).

Due to its favorable physic-mechanical activities, Si alleviates the adverse effects of water shortage and improves plant performance (Rady et al., 2019; Desoky et al., 2020). Nano-materials have emerged as a promising solution to various technological and environmental problems in several disciplines (Ansari and Husain, 2012). Compared with bulk Si, Si-NPs have a larger surface area with higher surface reactivity and solubility (Qados and Moftah, 2015). Specifically, particle size is a crucial factor influencing particle adhesion, absorption, and transportation in plant cells (Wang et al., 2009). Furthermore, NPs interact with plant cells by aiding the movement of numerous compounds that control plant metabolism and various physiological processes (Giraldo et al., 2014; Desoky et al., 2021).

However, knowledge of the effect and interaction of CCPB and Si-NPs on the performance of wheat plants cultivated in sandy soils under semi-arid environmental conditions is limited. Therefore, this study investigated the mechanism of inoculating sandy soil with CCPB and foliar application of Si-NPs to plants in influencing the physio-biochemical characteristics, performance, and antioxidative defenses of wheat grown under semi-arid environmental conditions. We hypothesized that inoculating soil with CCPB or foliar spraying with Si-NPs would improve wheat performance and defense against erosion in sandy soils under semi-arid environmental conditions. However, the co-addition of CCPB and Si-NPs would be more effective than single additions.

## Materials and methods

### Isolation and identification of calcium carbonate-precipitating bacteria

Calcium carbonate-precipitating bacterial isolates were isolated from the calcareous soil of the Mariout sector, Alexandria, Egypt. The soil sample was suspended in a sterilized saline solution (0.85% NaCl), and serial dilutions were carried out up to 10^–6^. Each dilution was plated on a medium containing 2.12 g NaHCO_3_, 3.0 g nutrient broth (Lab M Limited, Lancashire, UK), 20.0 g urea, 10.0 g NH_4_Cl, 30.0 mM CaCl_2_, 20.0 g agar L^–1^, and pH 8.5. The plates were then incubated at 28°C for 7 d. After isolation, all colonies were individually plated on CaCO_3_ precipitation medium supplemented with five concentrations of CaCO_3_ (10.0%, 15.0%, 20.0%, 25.0%, and 30.0%). Individual colonies that are found to be positive were selected based on their crystal formation visibility and purification by streaking on CaCO_3_ precipitation media without CaCl_2_.

The selected colonies were assessed under a stereomicroscope and primarily identified using Bergey’s manual of systematic bacteriology morphological and biochemical tests (Vos et al., 2009; Guinebretière et al., 2013). Further identification was performed by matrix-assisted laser desorption/ionization-time of flight (MALDI-TOF) mass spectrometry (MS) (Bruker Daltonics, Bremen, Germany) according to Schumaker et al. (2012) and Sauget et al. (2017). The manufacturers suggested score values of 2.30–3.00, 2.00–2.30, and 1.70–2.00 as highly probable species identification, secure genus identification and probable species identification, and probable genus identification, respectively.

### Optimization of calcium carbonate-precipitating bacterial isolates

#### Effect of pH on bacterial growth

The chosen isolates were inoculated into 10 ml nutrient broth tubes with different pH levels ranging from 1 to 14. The pH was adjusted using 1 N NaOH and 1 N HCl. The turbidity of each isolate was adjusted to the 0.5% McFarland standard, and the tubes were incubated for 24 h at 37°C. The growth was next assessed using a spectrophotometer (UV-2101/3101 PC; Shimadzu Corporation, Analytical Instruments Division, Kyoto, Japan) at an optical density of (OD)_600 nm_, and the results were compared with a bacterial blank suspension. Results were determined after 30 min, 1 h, 2 h, 4 h, 8 h, 24 h, and 32 h of inoculation.

#### Effect of temperature on bacterial growth

The selected bacterial isolates were briefly inoculated into 10 ml nutrient broth tubes, and the turbidity of each isolate was adjusted to the 0.5% McFarland standard. The tubes were incubated at 0, 10, 20, 30, 40, 50, and 60°C for 24 h. The growth was next assessed using a spectrophotometer (Shimadzu Corporation) at OD_600 nm_, and the results were compared with a bacterial blank suspension.

#### Production of urease

In assaying the urease activity, urea agar media (UAM) containing 15.0 g, 20.0 g, 1.0 g, 1.0 g, 5.0 g, 2.0 g, and 0.012 g of agar, urea, dextrose, pancreatic digest gelatin, sodium chloride, monosodium phosphate, and phenol red, respectively, were used. The medium pH was adjusted to a pH of 6.8 (Hammes et al., 2003; Chahal et al., 2011). Each candidate strain’s cell suspension (10^6^ cells mL^–1^) was inoculated on UAM. The plates were incubated for 24–48 h at 28°C, and the color change from yellow to pink was determined. Urease activity was measured as the concentration of the produced ammonium ions (NH^4 +^) as described by Tavares et al. (2021).

#### Calcium carbonate precipitation ability

All isolates were cultivated aerobically in 500 ml Erlenmeyer flasks with 100 ml of liquid CaCO_3_ precipitation medium. Flaks were incubated at 28°C for 3 d for CaCO_3_ precipitation and collection. The uninoculated liquid CaCO_3_ precipitation medium served as the control. After incubation, the entire culture was centrifuged for 1 min at 10,000 × *g*. The pellet was resuspended in a 50 ml TE buffer, which contained CaCO_3_ and bacterial cells (10 mM Tris, 1 mM EDTA at pH 8.5).

To digest the bacterial cell wall, lysozyme was added to the cell suspension at a final rate of 1 mg mL^–1^, and the tubes were incubated at 37°C for 1 h. Notably, centrifugation was used to remove the cell debris, and sterile distilled water (pH 8.5) was used to wash the pellet before being air-dried at 37°C for 24 h. The pellet was weighed to calculate the number of carbonate crystals precipitated by the various isolates.

### Experimental layout

A 2-year pot trial was performed in 2019/2020 and 2020/2021 using an open greenhouse at the Botany Department, Faculty of Agriculture, Zagazig University, Zagazig, Egypt. The average daily temperature was 17.7°C ± 2.0°C (15.3°C to 20.1°C), and the average daily relative humidity was 48.2 ± 4.3% (45.4%–51.0%). Wheat (*Triticum aestivum* L., cv. Misr 2) grains were obtained from the Agronomy Research Institute of the Agriculture Research Centre, Giza, Egypt. Before sowing, the grains were surface-sterilized for 5 min with 1% (v/v) sodium hypochlorite, washed several times with distilled water, and finally, air-dried for 1 h. Additionally, 10 kg of sandy soil was filled into plastic pots with inner diameters of 35 cm and depths of 30 cm. The physicochemical attributes of the tested soil, were measured (Page et al., 1982; Klute and Dirksen, 1986), and are illustrated in Supplementary Table 1.

A total of 240 pots were used in this study with the following investigated treatments: control, spraying plants with 1.00 or 1.50 mM Si-NPs; soil inoculated with *Bacillus lichenforms* (MA16), *Bacillus megaterium* (MA27), *Bacillus subtilis* (MA34), MA16 + 1.00 or 1.50 mM Si-NPs, MA27 + 1.00 or 1.50 mM Si-NPs, and MA34 + 1.00 or 1.50 mM Si-NPs. The recommended dose of inorganic nitrogen (N) as ammonium sulfate (205 g N kg^–1^ fertilizer) was added to all pots in three equal splits at a rate of 100 mg N kg^–1^ soil. The first split was added before the first irrigation, while the second and third doses were added 40 and 70 days after the first split.

Before sowing, phosphorus (P) and potassium (K) were applied to all experimental treatments at the recommended rates. Phosphorus was added as ordinary superphosphate at 15 mg P kg^–1^ soil, and K was applied as potassium sulfate at 40 mg K kg^–1^ soil. All pots were rotated (moved from one location to another) every 2 d to ensure equal light distribution and sunlight intensity to all plants. Notably, ten homogeneous grains were sown in each pot, leaving only five uniform seedlings in each pot after germination.

### Foliar application of silicon nanoparticles

Nano-Si dioxide was employed at 99.5% purity, 20–30 nm, and a surface area of 180–600 m^2^ g^–1^. A pressurized spray bottle was used to apply foliar sprays of 1 and 1.5 mM Si-NPs. In total, 0.1% of tween 20 was used as a surfactant (Desoky et al., 2021).

### Growth characteristics and yield determination

Wheat plants were harvested during each growing season to measure the growth attributes, physiology and biochemistry, and antioxidant defense system components after 65 days of planting. The leaf area (cm^2^) and plant height (cm) were determined. In measuring the dry weight (DW), samples were dried at 70°C until a constant weight was reached. During harvesting, the 1000 grain weight (g), the number of grains spike^−1^, and DW of grains plant^−1^ (g) were determined.

### Determination of physio-chemical constituents

The acetone extraction method was used to determine the contents of photosynthetic pigments—carotenoids and total chlorophylls (Arnon, 1949). Absorbance readings at 663 nm, 645 nm, and 480 nm were taken using a spectrophotometer (Shimadzu Corporation) to compute pigment content in mg g^–1^ leaf fresh weight. In upper fully expanded leaf tissue (second fully expanded leaf), chlorophyll fluorescence parameters using a PAM chlorophyll fluorimeter, the conductance of stomata (gs), net photosynthesis rate (*Pn*), and transpiration (*Tr*) rate were measured (Li et al., 2007). The formulas of Maxwell and Johnson (2000) were used to compute the maximum PS II *F*v/*F*m quantum yield as follows:

*F*v/*F*m = (*F*m–*F*0)/*F*m. Where, *F*v; variable fluorescence, *F*m; maximum light-adaptive fluorescence, and *F*0; minimum-adaptive fluorescence.

Photochemical quenching (qP) and non-photochemical quenching (NPQ) were determined as described by Han et al. (2022) and Ruban and Wilson (2021), respectively. Barrs and Weatherley’s (1962) method was used to measure the relative water content (RWC). We also determined the membrane stability index (MSI) based on the method reported by Rady (2011). The total inorganic ions that leached from the leaves (electrolyte leakage, EL) and malondialdehyde (MDA) contents were estimated according to the methods used previously (Heath and Packer, 1968; Sullivan and Ross, 1979). The leaf contents of superoxide oxide radical (O_2_^⋅–^; at A580 g^–1^ FW) and hydrogen peroxide (H_2_O_2_; μmol g^–1^ FW) were assessed following the procedures of Mukherjee and Choudhuri (1983) and Kubiś (2008), respectively. Proline (Pro) accumulation in leaves and total soluble sugar (TSS) content were also determined (Bates et al., 1973; Irigoyen et al., 1992). Additionally, glycine betaine (GB; Grieve and Grattan, 1983), and α-tocopherol (α-TOC; Ching and Mohamed, 2001) were calculated.

### Determination of enzymatic and non-enzymatic antioxidant compounds and activity

The contents (mol g^–1^ fresh weight) of ascorbate (AsA) and reduced glutathione (GSH) were assessed according to the methods of Griffith (1980) and Kampfenkel et al. (1995), respectively. Enzyme concentrations were extracted according to Vitória et al. (2001). The catalase (CAT) enzyme concentration was measured spectrophotochemically according to Chance and Maehly (1955), and peroxidase (POX) activity was estimated according to Thomas et al. (1982).

Ascorbate peroxidase (APX) was also determined spectrophotochemically (Fielding and Hall, 1978). Additionally, superoxide dismutase (SOD) activity was measured using the method of Sairam et al. (2002) by recording the drop in the absorbance of the superoxide-nitro blue tetrazolium complex by the enzyme. The glutathione reductase activity (GR; A564 min^–1^ mg^–1^ protein) was estimated according to Rao et al. (1996) after monitoring NADPH oxidation for three absorbances obtained at 340 nm.

### Statistical analysis

Data are shown as means ± SE. The experiments were designed using a completely randomized block design. We analyzed the data statistically using analysis of variance (ANOVA) and Tukey’s HSD test using SPSS 14.0 (SPSS Chicago, IL, USA) to analyze the significant differences between treatments (*p* ≤ 0.05).

## Results

### Identification and description of calcium carbonate-precipitating bacterial isolates and their activities

In total, 140 isolates were successfully isolated from calcareous soil on CCPB medium. Additionally, 34 isolates coded MA1–MA34 were grown on the CCPB plates supplemented with 10% of CaCO_3_, 26 isolates were grown on the CCPB plates supplemented with 15% of CaCO_3_, and 11 isolates survived at 20% of CaCO_3_. On the other hand, only four isolates grew on CCPB medium supplemented with 25% CaCO_3_. Only three isolates (MA16, MA27, and MA34) precipitated 30% CaCO_3_ (Table 1), and only these three isolates were selected for the experiments described below. Furthermore, the screened isolates were all gram-negative, aerobic, motile, and non-spore-forming bacilli. According to Bergey’s manual of systematic bacteriology morphological and biochemical tests, the obtained results showed that MA16, MA27, and MA34 isolates were similar to the *Bacillus* species.

**TABLE 1:** Isolation and screening of calcium carbonate precipitating bacteria (CCPB).

Isolate	CaCO_3_ concentration (%)				
	**10**	**15**	**20**	**25**	**30**
MA1	+	–	–	–	–
MA2	+	–	–	–	–
MA3	+	+	+	+	–
MA4	+	+	+	–	–
MA5	+	+	–	–	–
MA6	+	+	+	–	–
MA7	+	+	+	–	–
MA8	+	+	+	–	–
MA9	+	+	–	–	–
MA10	+	+	–	–	–
MA11	+	+	–	–	–
MA12	+	+	+	–	–
MA13	+	+	–	–	–
MA14	+	+	–	–	–
MA15	+	+	–	–	–
MA16	+	+	+	+	+
MA17	+	–	–	–	–
MA18	+	+	–	–	–
MA19	+	–	–	–	–
MA20	+	+	–	–	–
MA21	+	+	–	–	–
MA22	+	+	+	–	–
MA23	+	+	+	–	–
MA24	+	+	–	–	–
MA25	+	–	–	–	–
MA26	+	–	–	–	–
MA27	+	+	+	+	+
MA28	+	+	–	–	–
MA29	+	–	–	–	–
MA30	+	–	–	–	–
MA31	+	+	–	–	–
MA32	+	+	–	–	–
MA33	+	+	–	–	–
MA34	+	+	+	+	+

CaCO_3_, calcium carbonate, (+) growth, (–) no growth, (MA) bacterial isolate code.

They were identified as *B. lichenforms* MA16, *B. megaterium* MA27, and *B. subtilis* MA34. These three *Bacillus* spp. were further identified by MALDI–TOF mass spectrometry, as recommended by Biswas and Rolain (2013). Our results showed that they were 99% similar to the numerous *Bacillus* spp. According to the MALDI–TOF, score values were 2.332, 2.361, and 2.318. The local bacterial isolates *B. lichenforms* MA16, *B. megaterium* MA27, and *B. subtilis* MA34 were similar to *B. lichenforms* DSM30243*^T^, B. megaterium* DSM76*^T^*, and *B. subtilis* ssp. *subtilis* DSM10*^T^*, respectively (Supplementary Table 2). *Bacillus subtilis* MA34 grew better when treated with a higher concentration of CaCO_3_, thus inducing the best growth (Table 1).

**FIGURE 1:**
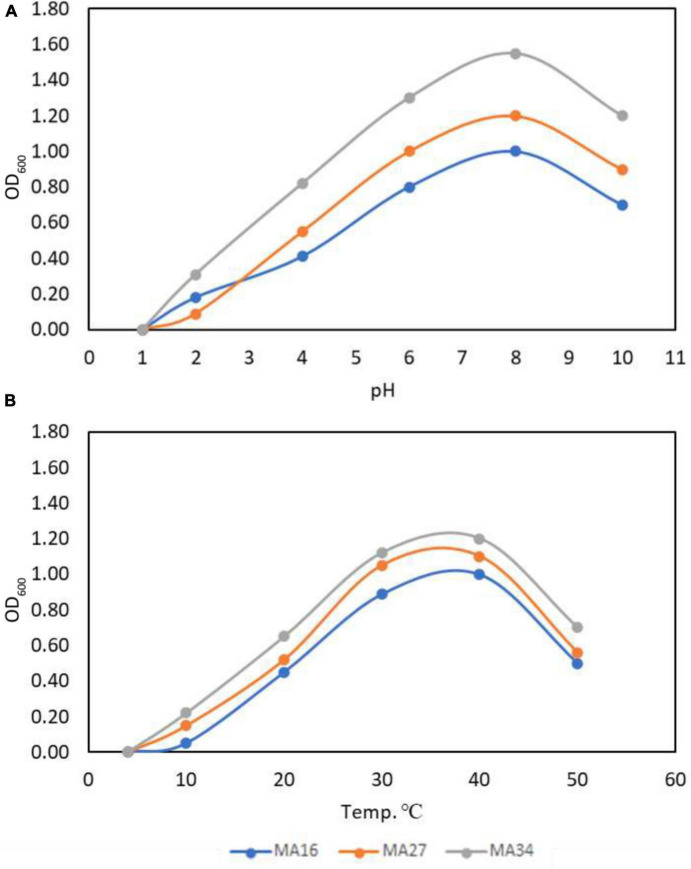
Optimization of calcium carbonate precipitating bacteria (CCPB). Effect of **(A)** pH and **(B)** temperature on *Bacillus lichenforms* (MA16), *Bacillus megaterium* (MA27), and *Bacillus subtilis* (MA34) growth.

Furthermore, the selected CCPB (MA16, MA27, and MA34) showed rapid growth and the highest turbidity at pH 8, indicating that these isolates are moderate alkaliphiles (Figure 1A). MA34 showed more growth at optimum pH with a turbidity of 1.5 × 10^6^ with a relative increase of 20% and 40% over MA27 and MA16, respectively. Conversely, the optimum temperature for the best growth of CCPB isolates was in the range of 30–40°C, with a preference for 37°C (Figure 1B). Similarly, the MA34 isolate had the best performance at 37°C.

**FIGURE 2:**
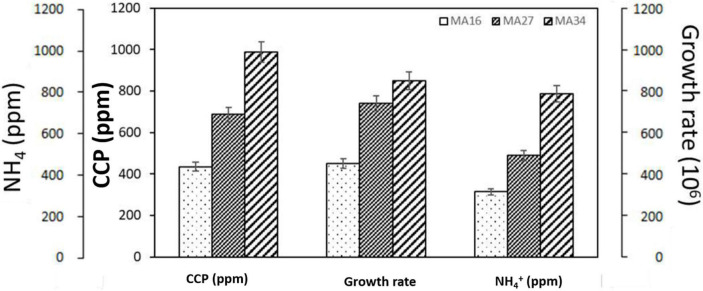
Calcium carbonate precipitation (CCP) and production of urease by the selected calcium carbonate precipitating bacteria, *Bacillus lichenforms* (MA16), *Bacillus megaterium* (MA27), and *Bacillus subtilis* (MA34). Urease activity was measured as the concentration of the produced ammonium ions (NH_4_^+^).

According to the precipitation mass per cell, *B. subtilis* MA34 was the most efficient strain in inducing CaCO_3_ precipitation. *B. subtilis* MA34 had the highest growth rate of 7.8 × 10^6^. They precipitated the highest value of CaCO_3_, *i.e.*, 990 ppm, with a relative increase of 25% and 57% for *B. megaterium* MA27 and *B. lichenforms* MA16, respectively (Figure 2).

Furthermore, the urease activity was recorded in all strains and expressed as the amount of NH_4_^+^. *B. subtilis* MA34 produced 775 ppm of NH_4_^+^ compared to 510 ppm and 325 ppm in *B. megaterium* MA27 and *B. lichenforms* MA16, respectively. Therefore, our findings showed that the mass of CaCO_3_ precipitation was directly and positively correlated with urease activity (Figure 2).

### Effects of calcium carbonate-precipitating bacteria and silicon nanoparticles on wheat growth and production

The soil inoculated with the three isolated CCPB (MA16, MA27, and MA34) and/or foliar application of wheat plants with Si-NPs at 1.0 and 1.5 mM significantly increased the plant height, leaf area, shoot DW, the 1000 grain weight, grain yield, and the number of grains spike^–1^ compared to control (Table 2). In addition, the combined treatments (CCPB + Si-NPs) were more effective than the individual applications (CCPB or Si-NPs) in improving the above attributes. Notably, MA34 + 1.5 mM Si-NPs was the best treatment, increasing plant height by 38.1% and 35.6%, shoot DW by 79% and 78.6%, leaf area by 50.5% and 42.7%, number of grains spike^–1^ by 110% and 116%, the 1000 grain weight by 43.4% and 49.1%, and plant grain yield by 173% and 186% in both seasons, respectively (Table 2).

**TABLE 2:** Effect of soil application with calcium carbonate precipitating bacteria (CCPB), *Bacillus lichenforms* (MA16), *Bacillus megaterium* (MA27), and *Bacillus subtilis* (MA34), and foliar application with silicon nanoparticles (Si-NPs) on growth and yield components of wheat plants (cv. Misr 2).

Treatment	Plant height (cm)	Shoot DW (g)	Leaf area (cm^2^)	Number of grains spike^–1^	1000 grain weight (g)	Grain yield plant^–1^ (g)
**First season**						
Control	60.1 ± 2.3i	3.39 ± 0.11l	19.2 ± 0.9h	20.1 ± 1.1k	36.8 ± 2.1i	3.54 ± 0.15j
1.0 mM Si-NPs	66.7 ± 2.5h	3.54 ± 0.12k	21.7 ± 1.1g	25.1 ± 1.6j	37.6 ± 2.22i	5.49 ± 0.21i
1.5 mM Si-NPs	69.2 ± 2.8g	3.71 ± 0.14j	22.9 ± 1.3f	28.0 ± 1.8i	39.6 ± 2.3h	6.17 ± 0.23h
MA16	75.4 ± 3.2e	4.16 ± 0.17h	25.6 ± 1.2d	31.7 ± 2.3g	43.8 ± 2.1f	7.14 ± 0.25f
MA27	73.8 ± 3.7f	3.97 ± 0.13i	24.6 ± 1.5e	29.5 ± 2.4h	42.1 ± 2.6g	6.67 ± 0.19g
MA34	77.6 ± 3.4d	4.44 ± 0.21g	25.7 ± 1.6d	34.2 ± 2.1f	45.9 ± 2.8e	7.42 ± 0.26f
MA16 + 1.0 mM Si-NPs	79.3 ± 3.4c	4.87 ± 0.22e	26.7 ± 1.8c	38.1 ± 2.6d	48.9 ± 2.4cd	8.72 ± 0.28d
MA16 + 1.5 mM Si-NPs	81.2 ± 3.9b	5.81 ± 0.25b	28.4 ± 1.7a	41.0 ± 1.9ab	51.8 ± 2.3a	9.85 ± 0.33ab
MA27 + 1.0 mM Si-NPs	78.3 ± 2.9d	4.63 ± 0.18f	26.3 ± 1.4c	36.5 ± 1.8e	47.8 ± 3.1d	8.30 ± 0.37e
MA27 + 1.5 mM Si-NPs	79.9 ± 2.8c	5.56 ± 0.23c	27.6 ± 1.6b	39.7 ± 2.2bc	50.4 ± 3.2b	9.59 ± 0.35b
MA34 + 1.0 mM Si-NPs	79.6 ± 3.5c	5.27 ± 0.31d	27.4 ± 1.9b	39.2 ± 2.3cd	49.9 ± 3.1bc	9.14 ± 0.38c
MA34 + 1.5 mM Si-NPs	83.0 ± 3.2a	6.07 ± 0.33a	28.9 ± 2.1a	42.3 ± 2.4a	52.8 ± 3.4a	9.69 ± 0.34a
**Second season**						
Control	62.2 ± 1.9i	3.51 ± 0.13l	20.6 ± 1.2i	19.2 ± 1.2i	37.3 ± 1.6j	3.60 ± 0.11h
1.0 mM Si-NPs	67.8 ± 2.8h	3.66 ± 0.15k	22.1 ± 1.3h	24.3 ± 1.3h	38.3 ± 1.8j	5.58 ± 0.19g
1.5 mM Si-NPs	70.3 ± 2.9g	3.83 ± 0.12j	23.2 ± 1.5g	27.3 ± 1.1g	40.4 ± 1.9i	6.33 ± 0.21f
MA16	76.8 ± 2.7e	4.36 ± 0.17h	26.1 ± 1.3e	30.9 ± 2.1f	44.6 ± 2.1g	7.40 ± 0.31d
MA27	75.1 ± 3.2f	4.17 ± 0.16i	25.1 ± 1.8f	28.4 ± 1.3g	42.8 ± 2.3h	6.89 ± 0.35e
MA34	79.1 ± 3.5d	4.64 ± 0.19g	26.2 ± 1.9e	34.2 ± 1.9e	47.2 ± 2.2f	7.64 ± 0.36d
MA16 + 1.0 mM Si-NPs	80.7 ± 3.4c	5.04 ± 0.25e	27.1 ± 2.1d	37.4 ± 2.4cd	49.7 ± 2.5de	8.91 ± 0.35c
MA16 + 1.5 mM Si-NPs	82.6 ± 3.6b	5.97 ± 0.24b	28.8 ± 2.2b	40.3 ± 2.6ab	52.7 ± 2.6b	10.1 ± 0.39a
MA27 + 1.0 mM Si-NPs	79.7 ± 3.5d	4.80 ± 0.21f	26.7 ± 2.3d	35.8 ± 1.6de	48.7 ± 2.7e	8.59 ± 0.28d
MA27 + 1.5 mM Si-NPs	81.2 ± 4.1c	5.73 ± 0.22c	28.1 ± 2.5c	39.0 ± 2.1bc	51.4 ± 2.6c	9.84 ± 0.29ab
MA34 + 1.0 mM Si-NPs	81.1 ± 3.8c	5.47 ± 0.26d	27.9 ± 2.3c	38.6 ± 2.9bc	50.7 ± 2.3cd	9.41 ± 0.34b
MA34 + 1.5 mM Si-NPs	84.4 ± 3.6a	6.27 ± 0.28a	29.4 ± 2.9a	41.5 ± 2.8a	55.6 ± 3.1a	10.3 ± 0.36a

Data are means ± SE. Within columns, values followed by different letters are significantly (*p* > 0.05) different according to Tukey’s HSD test. DW, dry weight.

### Effect of calcium carbonate-precipitating bacteria and silicon nanoparticles on gas exchange parameters and photosynthetic pigments in wheat plants

Compared with the control, the soil inoculated with the three isolated CCPB (MA16, MA27, and MA34) and/or foliar application of wheat plants with Si-NPs at 1 and 1.5 mM impacted significant increases in the chlorophylls, carotenoids, *Pn*, *Tr*, and *gs* and photosynthetic efficiency (quantum yield of PSII; FPSII, qP and efficiency of PSII; Fv/Fm) except for the NPQ, which was significantly reduced (Tables 3, 4). The combined addition of CCPB and Si-NPs was more effective than individual applications in improving these parameters. Additionally, MA34 + 1.5 mM Si-NPs showed the best treatment by increasing the total chlorophylls (61.2% and 63.5%), total carotenoids (18.3% and 23.3%), *Pn* (60.1% and 61.4%), *Tr* (55.6% and 52.8%), *gs* (69.9% and 62.1%), FPSII (118% and 124%), qP (49.5% and 50.4%), and Fv/Fm (64.1% and 57%); however, decreased NPQ by 48.9% and 49.7% in both seasons, respectively (Tables 3, 4).

**TABLE 3:** Effect of soil application with calcium carbonate precipitating bacteria (CCPB), *Bacillus lichenforms* (MA16), *Bacillus megaterium* (MA27), and *Bacillus subtilis* (MA34), and foliar application with silicon nanoparticles (Si-NPs) on photosynthetic pigments and gas exchange of wheat plants (cv. Misr 2).

Treatment	Total chlorophylls (mg g^–1^ FW)	Total carotenoids (mg g^–1^ FW)	Net photosynthetic rate; *Pn* (μ mol CO_2_ m^–2^ s^–1^)	Transpiration rate; *Tr* (mMol H_2_O m^–2^ s^–1^)	Stomatal conductance; *gs* (mMol H_2_O m^–2^ s^–1^)
	**First season**				
Control	1.55 ± 0.07l	0.763 ± 0.03g	7.18 ± 0.32l	4.13 ± 0.16j	0.343 ± 0.01j
1.0 mM Si-NPs	1.66 ± 0.06k	0.786 ± 0.02f	8.62 ± 0.33k	4.58 ± 0.15i	0.386 ± 0.02i
1.5 mM Si-NPs	1.76 ± 0.08j	0.796 ± 0.04f	8.80 ± 0.35j	4.72 ± 0.21h	0.410 ± 0.02h
MA16	1.86 ± 0.09h	0.816 ± 0.05e	9.28 ± 0.41h	5.31 ± 0.11f	0.460 ± 0.03f
MA27	1.82 ± 0.11i	0.810 ± 0.06e	9.15 ± 0.45i	5.16 ± 0.19g	0.433 ± 0.02g
MA34	1.94 ± 0.09g	0.836 ± 0.05d	9.54 ± 0.49g	5.48 ± 0.22e	0.476 ± 0.03e
MA16 + 1.0 mM Si-NPs	2.11 ± 0.12e	0.853 ± 0.04c	10.0 ± 0.49e	5.94 ± 0.23d	0.506 ± 0.03d
MA16 + 1.5 mM Si-NPs	2.43 ± 0.15b	0.890 ± 0.06b	11.2 ± 0.61b	6.25 ± 0.28b	0.573 ± 0.04a
MA27 + 1.0 mM Si-NPs	1.98 ± 0.09f	0.836 ± 0.08d	9.91 ± 0.52f	5.88 ± 0.21d	0.493 ± 0.03d
MA27 + 1.5 mM Si-NPs	2.35 ± 0.13c	0.886 ± 0.06b	11.0 ± 0.73c	6.12 ± 0.32c	0.553 ± 0.04b
MA34 + 1.0 mM Si-NPs	2.25 ± 0.14d	0.863 ± 0.07c	10.6 ± 0.62d	6.07 ± 0.33c	0.526 ± 0.03c
MA34 + 1.5 mM Si-NPs	2.50 ± 0.16a	0.903 ± 0.09a	11.5 ± 0.66a	6.43 ± 0.35a	0.583 ± 0.02a
	**Second season**				
Control	1.59 ± 0.08h	0.783 ± 0.04h	7.31 ± 0.41h	4.26 ± 0.18e	0.366 ± 0.02g
1.0 mM Si-NPs	1.73 ± 0.07g	0.801 ± 0.06g	8.77 ± 0.48g	4.73 ± 0.16d	0.423 ± 0.01f
1.5 mM Si-NPs	1.84 ± 0.09f	0.820 ± 0.07f	8.98 ± 0.52fg	4.90 ± 0.19d	0.446 ± 0.03f
MA16	1.91 ± 0.08f	0.870 ± 0.06cde	9.20 ± 0.59ef	5.53 ± 0.25c	0.503 ± 0.03de
MA27	1.89 ± 0.11f	0.856 ± 0.05de	9.11 ± 0.63efg	5.44 ± 0.23c	0.490 ± 0.02e
MA34	2.00 ± 0.12e	0.870 ± 0.04cde	9.46 ± 0.67e	5.70 ± 0.24c	0.516 ± 0.04cde
MA16 + 1.0 mM Si-NPs	2.18 ± 0.14d	0.886 ± 0.08cd	10.3 ± 0.69d	6.22 ± 0.29ab	0.540 ± 0.04c
MA16 + 1.5 mM Si-NPs	2.55 ± 0.15a	0.960 ± 0.06ab	11.5 ± 0.74ab	6.42 ± 0.29a	0.616 ± 0.05ab
MA27 + 1.0 mM Si-NPs	2.05 ± 0.13e	0.873 ± 0.07cde	10.1 ± 0.72d	6.05 ± 0.28b	0.530 ± 0.04cd
MA27 + 1.5 mM Si-NPs	2.44 ± 0.16b	0.960 ± 0.06ab	11.3 ± 0.78b	6.40 ± 0.32a	0.586 ± 0.03b
MA34 + 1.0 mM Si-NPs	2.34 ± 0.13c	0.910 ± 0.08bc	10.8 ± 0.71c	6.38 ± 0.31a	0.586 ± 0.02b
MA34 + 1.5 mM Si-NPs	2.60 ± 0.15a	0.966 ± 0.07a	11.8 ± 0.88a	6.51 ± 0.37a	0.630 ± 0.04a

Data are means ± SE. Within columns, values followed by different letters are significantly (*P* > 0.05) different according to Tukey’s HSD test.

**TABLE 4:** Effect of soil application with calcium carbonate precipitating bacteria (CCPB), *Bacillus lichenforms* (MA16), *Bacillus megaterium* (MA27), and *Bacillus subtilis* (MA34), and foliar application with silicon nanoparticles (Si-NPs) on chlorophyll fluorescence parameters, RWC and MSI of wheat plants (cv. Misr 2).

Treatment	FPSII	qP	NPQ	Fv/Fm	RWC (%)	MSI (%)
**First season**						
Control	0.420 ± 0.02i	6.10 ± 0.12k	1.20 ± 0.09a	0.546 ± 0.03h	60.4 ± 2.2i	40.7 ± 1.2k
1.0 mM Si-NPs	0.533 ± 0.04h	7.20 ± 0.21j	0.990 ± 0.08b	0.616 ± 0.02g	64.8 ± 2.3h	43.3 ± 1.5j
1.5 mM Si-NPs	0.586 ± 0.03g	7.48 ± 0.22i	0.956 ± 0.08bc	0.640 ± 0.04g	66.73.1 ± g	44.4 ± 1.4i
MA16	0.756 ± 0.05e	8.16 ± 0.23g	0.903 ± 0.07de	0.706 ± 0.05ef	70.4 ± 3.3e	47.9 ± 2.1g
MA27	0.710 ± 0.05f	7.86 ± 0.25h	0.930 ± 0.09cd	0.693 ± 0.04f	68.9 ± 3.5f	46.7 ± 2.6h
MA34	0.783 ± 0.06de	8.46 ± 0.28f	0.863 ± 0.04ef	0.746 ± 0.05e	71.7 ± 3.6d	49.5 ± 2.5f
MA16 + 1.0 mM Si-NPs	0.830 ± 0.06c	8.78 ± 0.23d	0.810 ± 0.05gh	0.823 ± 0.06cd	73.6 ± 3.8c	52.1 ± 2.9d
MA16 + 1.5 mM Si-NPs	0.893 ± 0.08b	9.01 ± 0.22ab	0.710 ± 0.03j	0.883 ± 0.04ab	76.5 ± 3.5ab	55.0 ± 1.9b
MA27 + 1.0 mM Si-NPs	0.796 ± 0.06d	8.66 ± 0.24e	0.836 ± 0.04fg	0.790 ± 0.05d	72.5 ± 2.9d	50.5 ± 2.8e
MA27 + 1.5 mM Si-NPs	0.863 ± 0.07b	8.94 ± 0.28bc	0.746 ± 0.02ij	0.860 ± 0.03abc	75.8 ± 3.4b	54.4 ± 2.9b
MA34 + 1.0 mM Si-NPs	0.850 ± 0.08bc	8.84 ± 0.26cd	0.786 ± 0.03hi	0.846 ± 0.06bc	74.2 ± 3.6c	53.5 ± 2.3c
MA34 + 1.5 mM Si-NPs	0.916 ± 0.07a	9.12 ± 0.31a	0.613 ± 0.02k	0.896 ± 0.04a	77.2 ± 3.8a	56.1 ± 2.5a
**Second season**						
Control	0.443 ± 0.02g	6.30 ± 0.14g	1.30 ± 0.08a	0.596 ± 0.02f	61.0 ± 3.2k	41.1 ± 2.4j
1.0 mM Si-NPs	0.560 ± 0.03f	7.43 ± 0.16f	1.05 ± 0.07b	0.666 ± 0.03e	65.3 ± 3.1j	43.8 ± 2.5i
1.5 mM Si-NPs	0.616 ± 0.04f	7.68 ± 0.19f	1.00 ± 0.06c	0.696 ± 0.04e	67.5 ± 3.6i	45.0 ± 2.9h
MA16	0.790 ± 0.04de	8.55 ± 0.22e	0.943 ± 0.07de	0.756 ± 0.05d	71.5 ± 3.8gh	48.5 ± 2.6g
MA27	0.746 ± 0.06e	8.23 ± 0.28e	0.986 ± 0.08cd	0.753 ± 0.05d	70.1 ± 3.4h	47.6 ± 3.2g
MA34	0.820 ± 0.07cd	8.76 ± 0.26d	0.913 ± 0.06ef	0.796 ± 0.04cd	72.2 ± 3.9fg	50.2 ± 2.5f
MA16 + 1.0 mM Si-NPs	0.890 ± 0.08b	9.16 ± 0.32bc	0.860 ± 0.05gh	0.883 ± 0.06b	74.5 ± 4.2de	52.9 ± 2.9d
MA16 + 1.5 mM Si-NPs	0.930 ± 0.08ab	9.44 ± 0.35ab	0.760 ± 0.03j	0.923 ± 0.07ab	77.3 ± 3.9ab	55.7 ± 2.8b
MA27 + 1.0 mM Si-NPs	0.863 ± 0.06bc	9.11 ± 0.34c	0.886 ± 0.04fg	0.830 ± 0.07c	73.4 ± 3.6ef	51.4 ± 3.1e
MA27 + 1.5 mM Si-NPs	0.910 ± 0.08b	9.30 ± 0.36abc	0.786 ± 0.04ij	0.906 ± 0.08ab	76.1 ± 3.8ab	55.4 ± 2.5bc
MA34 + 1.0 mM Si-NPs	0.896 ± 0.06b	9.20 ± 0.33abc	0.826 ± 0.05hi	0.896 ± 0.06ab	75.2 ± 3.7cd	54.4 ± 2.3c
MA34 + 1.5 mM Si-NPs	0.996 ± 0.08a	9.48 ± 0.32a	0.653 ± 0.02k	0.936 ± 0.09a	78.2 ± 3.5a	56.8 ± 2.9a

Data are means ± SE. Within columns, values followed by different letters are significantly (*P* > 0.05) different according to Tukey’s HSD test. RWC, relative water content; MSI, membrane stability index; PSII, photosystem II; FPSII, the quantum yield of PSII; qP, photochemical quenching; NPQ, non-photochemical quenching; Fv/Fm, the efficiency of PSII.

### Effect of calcium carbonate-precipitating bacteria and silicon nanoparticles on oxidative stress biomarkers and cell membranes in wheat plants

Applying three isolated bacteria and/or Si-NPs at different rates considerably increased the RWC and MSI but reduced EL, MDA, O_2_^⋅–^, and H_2_O_2_ in wheat plants compared with the control (Tables 4, 5). Furthermore, the co-addition of CCPB and Si-NPs was more effective than the individual application. Notably, MA34 + 1.5 mM Si-NPs were more effective than other treatments, increasing RWC by 27.8% and 28.1% and MSI by 37.8% and 38.1%, and decreasing El by 42.7% and 45.2%, MDA by 67.6% and 73.5%, O_2_^⋅–^ by 65% and 72.5%, and H_2_O_2_ by 71.3% and 77% in both seasons, respectively (Tables 4, 5).

**TABLE 5:** Effect of soil application with calcium carbonate precipitating bacteria (CCPB), *Bacillus lichenforms* (MA16), *Bacillus megaterium* (MA27), and *Bacillus subtilis* (MA34), and foliar application with silicon nanoparticles (Si-NPs) on oxidative stress of wheat plants (cv. Misr 2).

Treatment	EL (%)	MDA (μ mol g^–1^ FW)	O_2_^–^ (A580 g^–1^ FW)	H_2_O_2_ (μ mol g^–1^ FW)
**First season**				
Control	12.4 ± 0.41a	4.26 ± 0.11a	0.580 ± 0.03a	5.20 ± 0.14a
1.0 mM Si-NPs	10.4 ± 0.32b	3.18 ± 0.16b	0.456 ± 0.02b	4.55 ± 0.16b
1.5 mM Si-NPs	9.93 ± 0.52c	2.91 ± 0.12b	0.410 ± 0.01c	4.26 ± 0.12c
MA16	8.72 ± 0.65d	2.45 ± 0.13cd	0.376 ± 0.01d	3.58 ± 0.11d
MA27	9.19 ± 0.32e	2.56 ± 0.14c	0.393 ± 0.01cd	3.77 ± 0.14d
MA34	8.11 ± 0.48f	2.36 ± 0.13cd	0.346 ± 0.02e	3.23 ± 0.16e
MA16 + 1.0 mM Si-NPs	7.84 ± 0.36fgh	1.96 ± 0.11e	0.303 ± 0.01f	2.82 ± 0.14f
MA16 + 1.5 mM Si-NPs	7.44 ± 0.48i	1.45 ± 0.12f	0.236 ± 0.01h	1.77 ± 0.08i
MA27 + 1.0 mM Si-NPs	7.95 ± 0.48fg	2.18 ± 0.16de	0.323 ± 0.02ef	3.08 ± 0.11e
MA27 + 1.5 mM Si-NPs	7.62 ± 0.49hi	1.56 ± 0.15f	0.270 ± 0.01g	2.02 ± 0.06h
MA34 + 1.0 mM Si-NPs	7.73 ± 0.43ghi	1.40 ± 0.14f	0.273 ± 0.01g	2.47 ± 0.07g
MA34 + 1.5 mM Si-NPs	7.10 ± 0.51j	1.38 ± 0.13f	0.203 ± 0.01i	1.49 ± 0.063j
**Second season**				
Control	12.6 ± 0.69a	4.52 ± 0.15a	0.630 ± 0.04a	5.49 ± 0.13a
1.0 mM Si-NPs	10.3 ± 0.68b	3.11 ± 0.13b	0.426 ± 0.03b	4.37 ± 0.12b
1.5 mM Si-NPs	9.63 ± 0.85c	2.78 ± 0.12bc	0.376 ± 0.02bc	4.02 ± 0.13b
MA16	8.19 ± 0.32de	2.30 ± 0.13d	0.343 ± 0.01cde	3.26 ± 0.12cd
MA27	8.78 ± 0.54d	2.37 ± 0.14cd	0.360 ± 0.02cd	3.45 ± 0.14c
MA34	7.74 ± 0.63ef	2.23 ± 0.15d	0.313 ± 0.02def	2.98 ± 0.11cde
MA16 + 1.0 mM Si-NPs	7.55 ± 0.45efg	1.77 ± 0.17ef	0.270 ± 0.01fgh	2.53 ± 0.13ef
MA16 + 1.5 mM Si-NPs	7.11 ± 0.74fg	1.23 ± 0.16g	0.210 ± 0.01ij	1.49 ± 0.09hi
MA27 + 1.0 mM Si-NPs	7.62 ± 0.63ef	2.07 ± 0.18de	0.296 ± 0.01efg	2.81 ± 0.08de
MA27 + 1.5 mM Si-NPs	7.20 ± 0.38fg	1.31 ± 0.12g	0.243 ± 0.00ghi	1.86 ± 0.07gh
MA34 + 1.0 mM Si-NPs	7.48 ± 0.33fg	1.38 ± 0.13fg	0.233 ± 0.01hi	2.25 ± 0.12fg
MA34 + 1.5 mM Si-NPs	6.90 ± 0.29g	1.19 ± 0.12g	0.173 ± 0.01j	1.26 ± 0.06i

Data are means ± SE. Within columns, values followed by different letters are significantly (*P* > 0.05) different according to Tukey’s HSD test. EL, electrolyte leakage; MDA, malondialdehyde; O_2_^–^, superoxide anion radical; H_2_O_2_, hydrogen peroxide.

### Effect of calcium carbonate-precipitating bacteria and silicon nanoparticles on non-enzymatic antioxidant compounds in wheat plants

Compared with the control, the addition of CCPB and/or Si-NPs significantly increased osmoprotectants (Pro, TSS, and GB), α-TOC, AsA, and GSH (Table 6). The treatments of CCPB + Si-NPs were more effective than individual applications (CCPB or Si-NPs). MA34 + 1.5 mM Si-NPs was more effective than other treatments as it increased Pro (39.3% and 38.6%), TSS (114% and 115%), GB (48.5% and 48.9%), α-TOC (76.9% and 80.5%), AsA (94.2% and 93.5%), and GSH (157% and 147%) during the two seasons, respectively (Table 6).

**TABLE 6:** Effect of soil application with calcium carbonate precipitating bacteria (CCPB), *Bacillus lichenforms* (MA16), *Bacillus megaterium* (MA27), and *Bacillus subtilis* (MA34), and foliar application with silicon nanoparticles (Si-NPs) on osmoprotectants contents (free Pro and TSS), α-TOC, AsA, GSH and glycine betaine of wheat plants (cv. Misr 2).

Treatments	Free pro (μ mol g^–1^ DW)	TSS (mg g^–1^ DW)	α–TOC (μ mol g^–1^ DW)	AsA (μ mol g^–1^ FW)	GSH (μ mol g^–1^ FW)	GB (μ g g^–1^ DW
**First season**						
Control	25.9 ± 2.1h	16.1 ± 1.1k	1.78 ± 0.08j	1.56 ± 0.06i	1.12 ± 0.07l	40.6 ± 2.2k
1.0 mM Si-NPs	27.8 ± 2.2g	18.5 ± 1.3j	2.04 ± 0.11i	1.99 ± 0.08h	1.66 ± 0.06k	44.6 ± 2.3j
1.5 mM Si-NPs	29.1 ± 2.6f	19.1 ± 1.5j	2.26 ± 0.12h	2.24 ± 0.11g	1.72 ± 0.04j	45.5 ± 2.2i
MA16	32.2 ± 2.3d	22.5 ± 1.6h	2.51 ± 0.13fg	2.44 ± 0.12e	1.86 ± 0.06h	48.6 ± 2.5g
MA27	31.1 ± 2.8e	21.1 ± 2.1i	2.43 ± 0.14g	2.36 ± 0.13f	1.77 ± 0.07i	46.6 ± 2.6h
MA34	32.9 ± 2.6c	24.4 ± 2.3g	2.57 ± 0.11f	2.48 ± 0.14e	1.92 ± 0.05g	49.9 ± 2.5f
MA16 + 1.0 mM Si-NPs	34.1 ± 2.9b	28.9 ± 2.6e	2.82 ± 0.15d	2.70 ± 0.17cd	2.35 ± 0.11e	55.7 ± 2.9d
MA16 + 1.5 mM Si-NPs	35.5 ± 3.1a	33.1 ± 2.8b	3.02 ± 0.13b	2.97 ± 0.19a	2.80 ± 0.12b	59.6 ± 2.8b
MA27 + 1.0 mM Si-NPs	33.4 ± 3.2c	26.6 ± 2.9f	2.72 ± 0.13e	2.64 ± 0.16d	2.13 ± 0.11f	53.1 ± 2.4e
MA27 + 1.5 mM Si-NPs	34.5 ± 3.3b	31.4 ± 2.6c	2.96 ± 0.15bc	2.87 ± 0.18b	2.63 ± 0.13c	57.8 ± 2.8c
MA34 + 1.0 mM Si-NPs	34.4 ± 2.5b	30.2 ± 2.8d	2.92 ± 0.14cd	2.76 ± 0.17c	2.45 ± 0.14d	57.7 ± 2.7c
MA34 + 1.5 mM Si-NPs	36.1 ± 2.7a	34.6 ± 2.4a	3.15 ± 0.18a	3.03 ± 0.16a	2.88 ± 0.15a	60.3 ± 2.3a
**Second season**						
Control	26.4 ± 2.2h	16.3 ± 1.3k	1.85 ± 0.09i	1.71 ± 0.08i	1.25 ± 0.06h	41.7 ± 1.9k
1.0 mM Si-NPs	28.3 ± 2.3g	18.8 ± 1.8j	2.17 ± 0.12h	2.19 ± 0.11h	1.77 ± 0.05g	45.7 ± 1.8j
1.5 mM Si-NPs	29.5 ± 2.5f	19.5 ± 1.6j	2.38 ± 0.14g	2.50 ± 0.15g	1.94 ± 0.07f	46.6 ± 2.3i
MA16	32.9 ± 2.8d	22.9 ± 1.9h	2.68 ± 0.16f	2.75 ± 0.17ef	2.10 ± 0.11ef	49.7 ± 2.5g
MA27	31.9 ± 2.6d	21.5 ± 1.8i	2.62 ± 0.12f	2.56 ± f0.15g	1.95 ± 0.12f	48.1 ± 2.9h
MA34	33.6 ± 2.9cd	24.8 ± 2.2g	2.71 ± 0.14f	2.82 ± 0.17de	2.14 ± 0.14e	51.2 ± 2.8f
MA16 + 1.0 mM Si-NPs	34.7 ± 2.4b	29.4 ± 1.9e	2.97 ± 0.15de	2.98 ± 0.18bcd	2.58 ± 0.12cd	57.4 ± 3.1d
MA16 + 1.5 mM Si-NPs	36.1 ± 2.8a	33.9 ± 2.6b	3.26 ± 0.16ab	3.27 ± 0.21a	3.09 ± 0.16a	61.3 ± 3.2b
MA27 + 1.0 mM Si-NPs	33.9 ± 2.6c	27.1 ± 2.7f	2.89 ± 0.14e	2.87 ± 0.16cde	2.43 ± 0.18d	54.8 ± 3.5e
MA27 + 1.5 mM Si-NPs	35.2 ± 3.1b	31.8 ± 2.6c	3.14 ± 0.19bc	3.06 ± 0.22b	2.76 ± 0.13b	59.5 ± 3.3c
MA34 + 1.0 mM Si-NPs	34.9 ± 2.9b	30.8 ± 2.4d	3.05 ± 0.18cd	3.04 ± 0.23bc	2.64 ± 0.15bc	59.4 ± 3.2c
MA34 + 1.5 mM Si-NPs	36.6 ± 2.5a	35.1 ± 2.9a	3.34 ± 0.13a	3.31 ± 0.24a	3.09 ± 0.22a	62.1 ± 3.3a

Data are means ± SE. Within columns, values followed by different letters are significantly (*P* > 0.05) different according to Tukey’s HSD test. Pro, proline; DW, dry weight; TSS, total soluble sugars; α-TOC, α-tocopherol; AsA, ascorbate; GSH, glutathione; GB, glycine betaine.

### Effect of calcium carbonate-precipitating bacteria and silicon nanoparticles on antioxidant enzyme activity of wheat plants

Compared with the control, the soil inoculated with CCPB and/or plant spraying Si-NPs showed a significant increase in the activities of POX, CAT, APX, SOD, and GR in wheat plants (Table 7). Compared with individual treatments, the co-addition of CCPB and Si-NPs was demonstrated to be more effective. MA34 + 1.5 mM Si-NPs was the best treatment, showing an increase in the activities of CAT (17.9% and 17.2%), POX (185% and 193%), APX (18.7% and 18.8%), SOD (123% and 115%), and GR (57% and 56.7%) in both seasons, respectively (Table 7).

**TABLE 7:** Effect of soil application with calcium carbonate precipitating bacteria (CCPB), *Bacillus lichenforms* (MA16), *Bacillus megaterium* (MA27), and *Bacillus subtilis* (MA34), and foliar application with silicon nanoparticles (Si-NPs) on antioxidant enzymes of wheat plants (cv. Misr 2).

Treatment	CAT	POX	APX	SOD	GR
	
	A564 min^–1^ mg^–1^ protein				
	**First season**				
Control	60.7 ± 2.6k	0.560 ± 0.03h	55.5 ± 2.5k	3.21 ± 0.11k	32.1 ± 1.5f
1.0 mM Si-NPs	62.7 ± 2.8j	0.880 ± 0.04g	57.7 ± 2.6j	4.26 ± 0.13j	42.6 ± 1.5f
1.5 mM Si-NPs	63.7 ± 3.2i	1.01 ± 0.06f	58.4 ± 2.4i	4.66 ± 0.15i	43.1 ± 1.6f
MA16	65.8 ± 3.1g	1.09 ± 0.05ef	59.7 ± 2.7g	5.94 ± 0.16g	44.2 ± 1.7e
MA27	64.7 ± 3.4h	1.06 ± 0.07ef	59.1 ± 2.3h	5.58 ± 0.17h	43.2 ± 1.6f
MA34	66.4 ± 3.3f	1.66 ± 0.06e	60.3 ± 2.9f	6.07 ± 0.13f	44.9 ± 1.8de
MA16 + 1.0 mM Si-NPs	68.2 ± 3.6d	1.42 ± 0.07cd	61.7 ± 2.7e	6.63 ± 0.19d	46.6 ± 1.6c
MA16 + 1.5 mM Si-NPs	70.7 ± 3.5b	1.53 ± 0.08ab	64.8 ± 2.6b	7.04 ± 0.21b	49.6 ± 2.2a
MA27 + 1.0 mM Si-NPs	67.3 ± 3.7d	1.33 ± 0.07d	61.4 ± 2.8e	6.27 ± 0.17e	45.8 ± 2.7cd
MA27 + 1.5 mM Si-NPs	69.9 ± 3.8c	1.49 ± 0.06abc	63.5 ± 2.7c	6.87 ± 0.13c	48.6 ± 2.6b
MA34 + 1.0 mM Si-NPs	69.5 ± 3.6c	1.45 ± 0.05bc	62.6 ± 2.3d	6.80 ± 0.18c	47.7 ± 2.7b
MA34 + 1.5 mM Si-NPs	71.6 ± 3.4a	1.60 ± 0.07a	65.9 ± 2.9a	7.18 ± 0.21a	50.4 ± 2.9a
**Second season**					
Control	61.6 ± 2.5i	0.590 ± 0.02f	56.1 ± 1.9h	3.37 ± 0.12e	32.6 ± 1.6f
1.0 mM Si-NPs	63.6 ± 2.8h	0.910 ± 0.05e	58.5 ± 2.4g	4.51 ± 0.14d	43.4 ± 1.3e
1.5 mM Si-NPs	64.5 ± 2.6g	1.15 ± 0.06d	59.2 ± 2.4g	4.84 ± 0.15d	43.9 ± 1.8e
MA16	66.9 ± 3.7e	1.22 ± 0.08d	60.4 ± 3.2f	6.27 ± 0.16bc	45.1 ± 1.8de
MA27	65.7 ± 3.6f	1.15 ± 0.09d	60.3 ± 3.4f	6.10 ± 0.17c	44.1 ± 2.2e
MA34	67.4 ± 3.8e	1.26 ± 0.07cd	61.2 ± 3.5e	6.46 ± 0.19bc	45.7 ± 2.6cde
MA16 + 1.0 mM Si-NPs	68.7 ± 3.6d	1.57 ± 0.06ab	62.1 ± 3.5d	6.99 ± 0.18a	48.4 ± 2.4abc
MA16 + 1.5 mM Si-NPs	70.3 ± 3.8c	1.69 ± 0.07a	65.6 ± 3.6b	7.11 ± 0.22a	50.7 ± 2.9a
MA27 + 1.0 mM Si-NPs	68.4 ± 3.5d	1.44 ± 0.09bc	62.1 ± 3.7d	6.52 ± 0.21b	47.7 ± 2.7bcd
MA27 + 1.5 mM Si-NPs	70.7 ± 3.8bc	1.66 ± 0.07a	64.1 ± 3.4c	7.08 ± 0.25a	49.9 ± 2.6ab
MA34 + 1.0 mM Si-NPs	71.3 ± 3.7b	1.60 ± 0.4ab	63.5 ± 2.9c	7.04 ± 0.26a	49.3 ± 2.5ab
MA34 + 1.5 mM Si-NPs	72.2 ± 2.9a	1.73 ± 0.09a	66.6 ± 2.8a	7.27 ± 0.28a	51.1 ± 2.9a

Data are means ± SE. Within columns, values followed by different letters are significantly (*P* > 0.05) different according to Tukey’s HSD test. CAT, catalase; POX, peroxidase; APX, ascorbate peroxidase; SOD, superoxide dismutase; GR, glutathione reductase.

## Discussion

Calcium carbonate precipitation is a type of bio-mineralization that frequently occurs in bacteria (Boquet et al., 1973). It can be accomplished through biologically controlled or induced mineralization mechanisms (Mann, 1995). However, CCPB consists mainly of induced mineralization (Zamarreño et al., 2009). Globally, several bacterial species participate in mineral carbonate precipitation in various conditions such as soils, oceans, saline lakes, and freshwaters. Mineralization induced by microbial metabolic activities raises the alkalinity of the medium, thus facilitating CaCO_3_ precipitation (Castanier et al., 1999). The most common metabolic activity is urea hydrolysis, predominantly found in many microorganisms, majorly catalyzed by urease enzymes (Mobley and Hausinger, 1989). Urea hydrolysis using the microbial urease enzyme produces CO_3_^2–^ and ammonia (NH_3_), which raises soil pH and CO_3_^2–^ content which reacts with Ca^2+^ and precipitates it as CaCO_3_ (De Muynck et al., 2010).

Calcium carbonate-precipitating bacterial inoculation in sandy soil is one of the significant determinants of soil fertility, resulting in improved plant growth and productivity. It boosts the productivity of sandy soil by enhancing its biological activity, available nutrient content, and soil quality (Chaparro-Acuña et al., 2018; Elrys et al., 2018). These benefits of CCPB contributed to the high growth and yield of wheat when combined with Si-NPs foliar application (Table 2). This high performance was attributed to the improved chlorophyll fluorescence parameters and photosynthetic pigments of a wheat leaf affected by Si-NPs or CCPB treatments, principally the integrative CCPB + Si-NPs treatment. In addition, photosynthetic leaf pigments such as chlorophylls and carotenoids exhibited crucial roles in plant photosynthesis by capturing solar energy to fix carbon dioxide (CO_2_) (Table 3). The chlorophyll fluorescence proportion depends on the amount of solar energy absorbed by the chlorophyll molecules and the photosynthetic apparatus efficiency. As a result, photosynthetic pigments and chlorophyll fluorescence are essential components of photosynthesis (Netto et al., 2005; Singh et al., 2017).

During the light reaction photosynthesis phase, most PSII excitation energy is converted into ATP and NADP(H), consumed in CO_2_ fixation, and photorespiration coupled in the dark reaction phase. When PSII is not excited, a small fraction is lost as fluorescence, whereas the excess energy is dissipated as heat (Maxwell and Johnson, 2000). The chlorophyll fluorescence yield is detectable and regularly linked with photochemistry changes related to total photochemical efficiency (Singh et al., 2017). Hence, the concurrent increase in photosynthetic pigments and chlorophyll fluorescence, including gas exchange parameters by the co-addition of CCPB and Si-NPs, offers insight into the PSII function (Tables 3, 4).

The measurements of the photosynthetic pigments and chlorophyll fluorescence in this study serve as indicators of plant health. Therefore, supplying plants with CCPB + Si-NPs preserves the leaf pigments and chlorophyll fluorescence content, which is positively correlated with wheat yields. Additionally, maintaining the efficiency of the antioxidant system components and PSII function (Tables 6, 7) contributed to wheat performance, coupled with the beneficial effects of CCPB and/or Si-NPs. Furthermore, preserving the antioxidant system components in CCPB and/or Si-NPs treatments aided cell membrane stabilization in terms of low EL and MDA in plants (Table 5).

Moreover, CCPB and/or Si-NPs (especially the integrative CCPB + Si-NPs treatment) supplied to plants led to better yield and growth, which is attributed to the enhanced translocation of the photosynthetic assimilates from leaf to spike. This result is linked to the ability of plants to improve the antioxidant defense system components (Tables 6, 7). Therefore, this enhanced plant’s performance in cell expansions and meristem activities due to the retention of a sufficient quantity of water under sandy soil conditions causing increased contents of osmoprotectants such as TSS, Pro, and GB (Table 6).

In addition, the application of CCPB and/or Si-NPs induced a rise in wheat growth and production (Table 2), correlated with increased photosynthetic efficiency, chlorophyll biosynthesis, and gas exchange (Tables 3, 4). There was also a decrease in H_2_O_2_ and O_2_^⋅–^accumulations. Furthermore, after treating the plants with CCPB and/or Si-NPs, the efficiency of photosynthesis is boosted by increasing the ameliorative influences on chlorophyll fluorescence attributes (e.g., exc, PSII, qP, and Fv/Fm) while reducing the NPQ. Therefore, we revealed that the photosynthetic efficiency elevation in this study largely depends on the protected functioning of the photosynthetic light reaction, which functionally concurred with the enzymes PSII and PSI.

Silicon and/or CCPB application reduced H_2_O_2_ and O_2_^⋅–^ accumulation, MDA (a lipid peroxidation marker), and EL in the plants grown in sandy soil (Table 5). In addition, plants provided with CCPB and/or Si-NPs significantly reduced the membrane MDA and EL, improving membrane integrity attributed to the positive influence of the treatments on antioxidant system component maintenance (Table 7) and low peroxidation rates (Table 5). Plants treated with CCPB and/or Si-NPs significantly increased antioxidant enzyme activity with the AsA and GSH contents, shielding them from high H_2_O_2_ and O_2_^⋅–^. Furthermore, SOD eliminated O_2_^⋅–^ radicals through ROS dismutation together with APX and CAT. Si-stimulated SOD upregulation influences the substrates H_2_O_2_ and O_2_^⋅–^ (Gong et al., 2003; Desoky et al., 2019). Therefore, this mechanism decreases toxic hydroxyl radical (OH^–^) formation (Singh and Prasad, 2014).

The accumulation of AsA and GSH initiated by CCPB and/or Si-NPs protects wheat plants from ROS-stimulated injuries. Additionally, CCPB and/or Si-NPs-induced upregulation of the ROS scavenging pathway components such as AsA, GR, APX, and GSH which improves plant tolerance mechanisms against oxidative damage. For example, wheat plants treated with CCPB and/or Si-NPs decreased ROS accumulation (H_2_O_2_ and O_2_^⋅–^) (Table 5) and increased protection of the photosynthetic pathways (Tables 3, 4), contributing to improved growth and yield productivity (Table 2). H_2_O_2_, a byproduct of O_2_^⋅–^ elimination by the activity of SOD, degrades in the ascorbate–glutathione cycle and cytoplasm by APX and CAT, respectively (Feierabend, 2005). Furthermore, the APX enzyme is critical in scavenging H_2_O_2_ in the chloroplasts and cytosol, averting H_2_O_2_ diffusion into other organelles that could potentially cause damage. Furthermore, the optimal operation of the AsA-GSH cycle pathway when plants were provided with CCPB and/or Si-NPs (Tables 6, 7) effectively preserved GSH and AsA components, reducing the oxidative stress (H_2_O_2_ and O_2_^⋅–^; Table 5). Therefore, the increased non-enzymatic and enzymatic antioxidant activity is linked to improved plant health (Elrys et al., 2020; El-Saadony et al., 2021).

Our study treated wheat plants with CCPB and/or Si-NPs, causing the accumulated osmoprotectants (e.g., TSS, GB, Pro, and α-TOC) to raise the RWC, MSI, and water content of plants in sandy soil (Table 4). We also reported limited Pro accumulation, as pro-synthesizing enzymes were upregulated, whereas the catabolizing enzymes were downregulated (Table 6). This result was due to increased antioxidant system components, GB, and TSS. Additionally, Pro was incorporated into proteins (Ahmad, 2010). Si-NPs increased TSS and GB accumulation, maintaining balanced plant water (Ahanger et al., 2014).

Conclusively, wheat plants treated with CCPB and/or Si-NPs developed some potential mechanisms in sandy soils. These include increased accumulation of osmoprotectant compounds that provide a mechanism for water loss reduction in leaves and boost their water content to maintain healthy metabolic processes and membrane stability under sandy soil conditions. Additionally, increased antioxidant activity (enzymatic and non-enzymatic) provides a potential mechanism for strengthening the antioxidant defense system and increasing plant resistance. These mechanisms, combined with others, resulted in plant leaves remaining green, delayed senescence, and enhanced photosynthesis efficiency and chlorophyll content to maintain healthy plants. Therefore, these improvements in antioxidant defense components help limit oxidative damage.

## Data availability statement

The original contributions presented in this study are included in the article/Supplementary material, further inquiries can be directed to the corresponding authors.

## Author contributions

E-SD, SA, KE-T, and ME-S conceived and designed the research. MR, SA, KE-T, and ME-S supervised the study. E-SD, MR, MN, NM, and AE performed open greenhouse experiments. E-SD, MR, and MN performed the microscopic experiments. E-SD, SA, KE-T, and ME-S analyzed the data. AE, AM, SA, and KE-T assisted with experiments and/or data evaluation. E-SD, SA, KE-T, and ME-S wrote the manuscript. All authors critically revised the manuscript and approved the final version.
