# Supplementary material for: Integrated application of bacterial carbonate precipitation and silicon nanoparticles enhances productivity, physiological attributes, and antioxidant defenses of wheat (Triticum aestivum L.) under semi-arid conditions
Source: Front Plant Sci. 2022 Oct 25;13:947949. doi: 10.3389/fpls.2022.947949 (PMC9641219; doi:10.3389/fpls.2022.947949)
Supplement: Supplementary file 1 [file Data_Sheet_1.pdf]

Supplementary material

Integrated Application of Bacterial Carbonate Precipitation and Silicon Nanoparticles Enhances Productivity, Physiological Attributes and Antioxidant Defenses of Wheat (*Triticum aestivum* L.) under Semi-arid Conditions

El-Sayed M. Desoky, Mostafa M. Rady, Maha M. Nader, Nadeen G. Mostafa, Ahmed S. Elrys, Archana Mathai, Synan F. AbuQamar*, Khaled A. El-Tarabily*, and Mohamed T. El-Saadony

*** Correspondence:**
Synan F. AbuQamar: sabuqamar@uaeu.ac.ae
Khaled A. El-Tarabily: ktarabily@uaeu.ac.ae

Supplementary material

Table S1. Physical and chemical properties of the experimental soil used for two different seasons before and after its inoculation with calcium carbonate-precipitating bacteria (CCPB).

Table S2. Identification of the calcium carbonate-precipitating bacteria (CCPB) using matrix-assisted laser desorption/ionization-time of flight (MALDI-TOF) mass spectrometry (MS).

Table S1. Physical and chemical properties of the experimental sandy soils used for two different seasons before and after its inoculation with calcium carbonate-precipitating bacteria **(**CCPB).

Parameter	Before soil inoculation with CCPB		After soil inoculation with CCPB	
	First season	Second season	First season	Second season
Soil EC (dS m^{-1})	0.35	0.39	0.41	0.52
Soil pH	8.11	8.19	7.94	7.92
$CaCO_3$ (g kg^{-1})	5.21	5.28	6.13	6.09
Soil CEC ($cmol_c$ kg^{-1})	5.68	5.59	6.72	6.46
Organic matter (g kg^{-1})	6.60	7.10	6.90	7.40
Nitrogen (mg kg^{-1} soil)	22.5	22.9	24.3	24.8
Phosphorus (mg kg^{-1} soil)	5.30	5.50	8.30	9.10
Potassium (mg kg^{-1} soil)	65.3	66.5	66.1	66.9

EC = Electrical conductivity

Table S2. Identification of the calcium carbonate-precipitating bacteria (CCPB), using matrix-assisted laser desorption/ionization-time of flight (MALDI-TOF) mass spectrometry (MS).

Isolate	Local isolate	Identified bacteria	Similarity	Score value
MA16	*Bacillus lichenforms*	*Bacillus lichenforms* DSM30243^{T}	99%	2.318
MA27	*Bacillus megaterium*	*Bacillus megaterium* DSM76^{T}	99%	2.361
MA34	*Bacillus subtilis*	*Bacillus subtilis* ssp. *subtilis* DSM10^{T} DSM	99%	2.332

This method was used according to Biswas and Rolain (2013) and Sauget et al. (2017).
